# Treatment of Pneumonitis

**Published:** 1874-03-15

**Authors:** A. Hermann

**Affiliations:** Pesth


### TREATMENT OF PNEUMONITIS.

By Dr. A. Hermann, of Pesth.

*Translated for* The Examiner, *from the Allgemeine Wiener Med. Zeitung, by* H. Gradle.

(Continued from Number IV.)

THE inquiry as to the influence of age on the inflammation of either lung, finds an answer in the following tables:

I.—Right Lung Affected.

| | | | | | | |
|---|---|---|---|---|---|---|
| Of | 1 | case at the age of | 9 | years, there were | no | deaths. |
| " | 16 | " " " " | 11-20 | " " " | " | " |
| " | 23 | " " " " | 21-30 | " " " | 1 | " |
| " | 11 | " " " " | 31-40 | " " " | 1 | " |
| " | 10 | " " " " | 41-50 | " " " | 2 | " |
| " | 9 | " " " " | 51-60 | " " " | 1 | " |
| " | 5 | " " " " | 61-70 | " " " | 4 | " |
| " | 4 | " " " " | 71-80 | " " " | 3 | " |
| | 79 | " | ----- | | 12 | " |

II.—Left Lung Affected.

| | | | | | | |
|---|---|---|---|---|---|---|
| Of | 17 | cases at the age of | 11-20 | years, | no | deaths. |
| " | 13 | " " " " " | 21-30 | " | 1 | " |
| " | 11 | " " " " " | 31-40 | " | no | " |
| " | 3 | " " " " " | 41-50 | " | " | " |
| " | 3 | " " " " " | 51-60 | " | 2 | " |
| " | 3 | " " " " " | 61-70 | " | no | " |
| " | 0 | " " " " " | 71-80 | " | " | " |
| " | 1 | " " " " " | -94 | " | " | " |
| | 51 | " | ----- | | 3 | " |

III.—Both Lungs Affected.

| | | | | | | |
|---|---|---|---|---|---|---|
| Of | 5 | cases at the age of | 11-20 | years, | no | deaths. |
| " | 5 | " " " " " | 21-30 | " | 2 | " |
| " | 4 | " " " " " | 31-40 | " | no | " |
| " | 1 | " " " " " | 41-50 | " | " | " |
| " | 2 | " " " " " | 51-60 | " | 2 | " |
| " | 2 | " " " " " | 61-70 | " | 1 | " |
| | 19 | " | ----- | | 5 | " |

Altering this table, for the sake of simplicity, the following results are obtained.

Of 61 cases of inflammation of the right lung, below 50 years, there were 4 deaths—a mortality of 6.5 per cent.

Of 18 cases of inflammation of the right lung, above 50 years, there were 8 deaths—a mortality of 44.4 per cent.

Of 44 cases of inflammation of the left lung, below 50 years, there was 1 death—a mortality of 2.2 per cent.

Of 7 cases of inflammation of the left lung, above 50 years, there were 2 deaths—a mortality of 28.6 per cent.

Of 15 cases of inflammation of both lungs, below 50 years, there were 2 deaths—a mortality of 13.3 per cent.

Of 4 cases of inflammation of both lungs, above 50 years, there were 3 deaths—a mortality of 75.0 per cent.

Further remarks on these statistics are superfluous. Their significance is self-evident; but their practical application is strikingly illustrated by the unequal success in different years, though the locality, the accommodations, the nursing, and even the treatment, were the same for all patients. Of the cases of genuine pneumonitis treated, there were—

| | | | | | | | |
|---|---|---|---|---|---|---|---|
| In | 1866, 7 | cases, with | 3 | deaths, or | 42.8 | per | cent. |
| " | 1867, 19 | " " | 1 | " " | 5.2 | " | " |
| " | 1868, 28 | " " | 2 | " " | 7.1 | " | " |
| " | 1869, 19 | " " | 2 | " " | 10.5 | " | " |
| " | 1870, 36 | " " | 8 | " " | 22.2 | " | " |
| " | 1871, 33 | " " | 6 | .. " | 18.1 | " | " |
| " | 1872, 21 | " " | 3 | " " | 14.2 | " | " |

As before stated, the surrounding circumstances were not altered during the entire period. The varying mortality, therefore, of 42.8 per cent. one year, followed by 5.2 per cent. the next, rising subsequently again to 22.2 per cent., can only be accounted for by the differing malignancy of the disease, and the age.

Unable to explain the difference in severity, physicians of former times escaped the ensuing dilemma by assumption of an arbitrary "Genius Epidemicus," a spirit which would now and then slight, and again favor, the individual practitioner—an ingenious little device, quite satisfactory in those times, and certainly as scientific as some expressions of our modern terminology. If, for instance, of a number of children of the same parents, equally reared and educated, one or two present the scrofulous diathesis, while the rest escape, the term, " Predisposition to scrofulosis " conveys, certainly, no more information than the ancients derived from a consultation of the " Genius Epidemicus." Critical observation, however, has rendered this evil spirit more tangible. The fanciful outgrowth of imagination, taking the place of patient investigation and deliberate reasoning, could not maintain its existence against the results of these better methods of research; and partially, at least, is the present generation familiar with the influences and conditions whose sum represents the "Genius Epidemicus." But to judge correctly of the import of each influence, circumstance, as age, sex, constitution, hygienic relations, previous health, etc., is a problem, the solution of which will differ with the subjective view of the physician, who, perhaps, unacquainted with, or underrating one, will comparatively overestimate the other; whence, the *tout ensemble*, in a prognostic aspect, will not be of the same significance with different observers. Therefore, when Juergensen speaks of a pneumonitis disappearing in twenty-four to thirty hours, the author, without any personal insinuations whatever, cannot but doubt such an occurrence—at least, denies having seen it; and, since he affirms that all doubtful cases have been rigorously excluded from his analysis, *can conscientiously maintain that no difference in the inherent malignancy of his cases existed.*

Paying due consideration to the age of the patient, which, alone, will not explain the great discrepancy of the results, another factor is to be sought in the extent of lesion, the involvement of one or more lobes, the affection of the right or left side, or both; and this theoretical explanation is sustained by the analysis below:

| | | | | | | | | |
|---|---|---|---|---|---|---|---|---|
| 1866— | 3 | cases | inflam'n | right | lung, | with | no | deaths. |
| " | 3 | " | " | both | " | " | 2 | " |
| " | 1 | " | " | (?)* | " | " | 1 | " |
| Tot'l, | 7 | " | | ----- | | " | 3—42.8 p.c. | |
| 1867— | 10 | cases | inflam'n | right | lung, | with | no | deaths. |
| " | 5 | " | " | left | " | " | " | " |
| " | 3 | " | " | both | " | " | 1 | " |
| " | 1 | " | " | (?)* | " | " | no | " |
| Tot'l, | 19 | " | | ----- | | " | 1— 5.2 p.c. | |
| 1868— | 11 | cases | inflam'n | right | lung, | with | 1 | death. |
| " | 9 | " | " | left | " | " | " | " |
| " | 4 | " | " | both | " | " | no | " |
| " | 4 | " | " | (?)* | " | " | 1 | " |
| Tot'l, | 28 | " | | ----- | | " | 2— 7.1 p.c. | |
| 1869— | 6 | cases | inflam'n | right | lung, | with | 1 | death. |
| " | 9 | " | " | left | " | " | 1 | " |
| " | 4 | " | " | both | " | " | no | " |
| Tot'l, | 19 | " | | ----- | | " | 2—10.5 p.c. | |
| 1870— | 23 | cases | inflam'n | right | lung, | with | 6 | deaths. |
| " | 10 | " | " | left | " | " | 1 | " |
| " | 3 | " | " | both | " | " | 1 | " |
| Tot'l, | 36 | " | | ----- | | " | 8—22.2 p.c. | |
| 1871— | 16 | cases | inflam'n | right | lung, | with | 2 | deaths. |
| " | 9 | " | " | left | " | " | no | " |
| " | 2 | " | " | both | " | " | 2 | " |
| " | 6 | " | " | (?)* | " | " | 2 | " |
| Tot'l, | 33 | " | | ----- | | " | 6—18.1 p.c. | |
| 1872— | 12 | cases | inflam'n | right | lung, | with | 2 | deaths. |
| " | 8 | " | " | left | " | " | 1 | " |
| " | 1 | " | " | both | " | " | no | " |
| Tot'l, | 21 | " | | ----- | | " | 3—14.2 p.c. | |

SUMMARY:

| | |
|---|---|
| Cases | 163 |
| Deaths | 25 |
| Mortality (per cent.) | 15.34 |

These facts throw a decidedly dubious light on the superiority of any treatment, if judged by the comparative mortality in a certain number of cases, unless they are completely specified and classified; for how are we to know whether or not the " Genius Epidemicus " did not extend its arbitrary partiality to the physician who records the analysis, and favor him with, perhaps, unusually light cases, or with individuals of tough

* Unknown which side affected.

constitution and great powers of endurance, to the detriment of his neighbor, whose ill-fate may have conducted him to the bedside of patients struggling in vain for life? But the great discrepancy in the mortality of different years finds no sufficiently satisfactory cause in the seat of the lesion. The author, therefore, cites the variation of the other factor referred to—the age:

| | | | | | | | | |
|---|---|---|---|---|---|---|---|---|
| 1866— | 3 | cases | below | 50 | years, | with | no | deaths. |
| " | 3 | " | above | 50 | " | " | 3 | " |
| " | 1 | " | unknown | | " | " | no | " |
| 1867— | 18 | " | below | 50 | " | " | 1 | " |
| " | 1 | " | above | 50 | " | " | no | " |
| 1868— | 23 | " | below | 50 | " | " | no | " |
| " | 3 | " | above | 50 | " | " | 2 | " |
| " | 2 | " | unknown | | " | " | no | " |
| 1869— | 16 | " | below | 50 | " | " | 1 | " |
| " | 3 | " | above | 50 | " | " | 1 | " |
| 1870— | 27 | " | below | 50 | " | " | 3 | " |
| " | 9 | " | above | 50 | " | " | 5 | " |
| 1871— | 24 | " | below | 50 | " | " | 1 | " |
| " | 9 | " | above | 50 | " | " | 5 | " |
| 1872— | 15 | " | below | 50 | " | " | no | " |
| " | 6 | " | above | 50 | " | " | 3 | " |

The therapeutic measures were not altered during the entire period; but had they been during any special year, the remedial triumph that might have been claimed in 1867-68, could not have been disputed by an unbiased judge; and only the knowledge obtained from a careful and extended series of statistics, would prevent a candid observer from falling into error in this direction.

Perhaps a more correct criterion of the success of any treatment, is to be found in the duration of the disease thus influenced; and, substituting for the length of time of the affection (which is frequently beyond accurate computation) the period of hospital residence, the author reports this as—

| | | | | | | | | |
|---|---|---|---|---|---|---|---|---|
| From | 1 | to | 7 | days | for | 6 | patients. |
| " | 8 | " | 14 | " | " | 47 | " |
| " | 15 | " | 21 | " | " | 46 | " |
| " | 22 | " | 28 | " | " | 18 | " |
| " | 29 | " | 35 | " | " | 14 | " |
| " | 36 | " | 42 | " | " | 4 | " |
| " | 43 | " | 45 | " | " | 3 | " |
| | | | | | Total, | 138 | " |

The average number of days of hospital residence is expressed by 18.37 days; and, since rarely a patient enters the building but that three times twenty-four hours have elapsed since the onset of the disease, twenty-one days can be taken as the average duration of pneumonitis.

Since age exercises such vast influence on the mortality, it might be profitable to study the extent of its sway on the duration of the disease; an answer to which inquiry is furnished in the following table:

| At age of 9 years. | 11–20 yrs. | 21–30 yrs. | 31–40 yrs. | 41–50 yrs. | 51–60 yrs. | 61–70 yrs. | 71–80 yrs. | –94 yrs. | Patients remained in the Hospital. |
|---|---|---|---|---|---|---|---|---|---|
| ---- | 2 | 1 | 2 | -- | 1 | -- | -- | -- | 1 to 7 days. |
| 1 | 17 | 16 | 5 | 4 | 2 | -- | 1 | -- | 8 " 14 " |
| ---- | 14 | 14 | 8 | 4 | 3 | 1 | -- | 1 | 15 " 21 " |
| ---- | 5 | 3 | 2 | 3 | 3 | 1 | -- | -- | 22 " 28 " |
| ---- | 1 | 3 | 7 | 1 | -- | 2 | -- | -- | 29 " 35 " |
| ---- | 2 | 1 | 1 | -- | -- | -- | -- | -- | 36 " 42 " |
| ---- | -- | 1 | 1 | 1 | -- | -- | -- | -- | 43 " 45 " |

If, for facility of survey, we limit our attention to the difference between patients above and below fifty years of age, we find that—

| Below 50 yrs. | Above 50 yrs. | Patients remained in the Hospital. |
|---|---|---|
| 5 | 1 | 1 to 7 days. |
| 43 | 3 | 8 " 14 " |
| 40 | 5 | 15 " 21 " |
| 13 | 4 | 22 " 28 " |
| 12 | 2 | 29 " 35 " |
| 4 | | 36 " 42 " |
| 3 | | 43 " 45 " |

Now, while the majority of all patients did not remain above the average number of days, of those above fifty years a much greater percentage necessitated a longer than the mean duration of treatment; and it is undoubtedly the experience of most practitioners, that the older the individual, the longer will he require medical attention. Neither is this contrary to the laws of nature; and it is thus seen that even this index of remedial efficacy, viz.: the duration of the disease, is liable to be misinterpreted, without due regard to all accompanying circumstances.

A slight doubt is perhaps admissible, whether the time of discharge was determined by an invariable guide. The author informs us that the completion of hospital residence was governed by the perfectly normal temperature, the absence of abnormal results by percussion and auscultation, and such a degree of physical and mental well-being of the patient as to induce a desire for a change of location; but even if a slight inaccuracy does exist in these cases, there is certainly none in the record of deaths. As before remarked, 25 patients out of 163 succumbed to the disease; and of these,

| | | | | | |
|---|---|---|---|---|---|
| 8 | died | 1 | to | 3 | days after admission to the hospital. |
| 9 | " | 4 | " | 7 | " " " " " " |
| 5 | " | 8 | " | 11 | " " " " " " |
| 3 | " | 12 | " | 16 | " " " " " " |

The first week, therefore, closed with 68 per cent. of all deaths; and as the mean duration of life, in these cases, was found to be 5.96 days, this analysis, as well as universal experience, justifies the physician in giving hope after the lapse of the first week. In examining the influence of age on the lease of life, in this affection, results like the following are obtained:

| Below 50 yrs. | Above 50 yrs. | Patients survived. |
|---|---|---|
| 1 | 7 | 1 to 3 days. |
| 3 | 6 | 4 " 7 " |
| 2 | 3 | 8 " 11 " |
| 1 | 2 | 12 " 16 " |

Or, in giving a *resume*: of patients below 50 years of age, 57.1 per cent. died the first week, and 42.8 per cent. the second; while of those above 50 years, 72.2 per cent. died the first week, and 27.7 the second.

The hints to be derived from these figures, for prognosis, are, *that the ratio of mortality in pneumonitis increases with the age; and that the older the patient, the sooner will he succumb*: since three-fourths of the fatal cases above fifty years of age did not survive the first week, and none the second; while but one-half of the younger victims of death failed in the first seven days, and a number only after the second week.

*(To be continued.)*

